# Morphological, Chemical, and Biological Investigation of Ionic Substituted, Pulse Current Deposited Calcium Phosphate Coatings

**DOI:** 10.3390/ma13204690

**Published:** 2020-10-21

**Authors:** Monika Furko, Csaba Balázsi

**Affiliations:** Institute for Technical Physics and Materials Science, Centre for Energy Research, Konkoly-Thege str. 29-33, 1121 Budapest, Hungary; balazsi.csaba@energia.mta.hu

**Keywords:** bioactive coatings, biodegradability, calcium phosphate, electrodeposition

## Abstract

Ionic substituted calcium phosphate coatings (iCP) have been prepared by the electrochemical pulse current deposition technique with an alternate pulse on and off time of 5 ms onto a titanium alloy substrate. The elemental distribution and morphology of the deposited layers have been extensively studied by scanning electron microscopy (SEM), energy dispersive X-ray analysis (EDX), and transmission electron microscopy (TEM). The crystallinity and phase structure of iCPs have been investigated by X-ray diffraction (XRD). The corrosion characteristics and biodegradability of coatings have been determined by electrochemical measurements, recording potentiodynamic curves in a physiological solution over a long-term immersion period. The cell viability tests confirmed that the iCP coating was biocompatible, while the corrosion tests proved its biodegradable characteristic. In our paper, we compare the morphological, chemical, and biological characteristics of silver and zinc substituted calcium phosphate layers deposited by the electrochemical method.

## 1. Introduction

In orthopaedic surgeries, the medical implant materials are key issues. In past decades, intensive research have been conducted to improve the implants’ surface that are in contact with human tissues [1]. The development of an appropriate implant design is to try to promote bone cell growth especially at the early stage of implantation. The surface of implant is the first that interacts with the human tissue; therefore, modifications of the surface are necessary to increase the biocompatible and osteoconductive characteristics of different medical materials [2,3].

Other crucial problems that might occur after implantation are the implant-related bacterial infections because they are difficult to treat [4]. As a consequence, implant removal, constructive surgeries, and long-term antibiotic treatment are needed without considering any adverse side effects [5,6,7,8,9]. In order to resolve these problems, metals and elements which possess an antimicrobial property, as well as the ability to promote new bone formation can be incorporated into the calcium phosphate phases. These elemental substitutions might modify the crystal structure and induce some changes in the characteristics of materials, such as phase stability, reactivity, surface characteristics, and they might change the bioactivity and biocompatibility [10].

Abundant numbers of the reports can be found in the scientific literature dealing with the preparation of calcium phosphate and ionic doped CP coatings onto metallic implant surfaces. We can distinguish physical methods (pulsed laser deposition, chemical vapour deposition, physical vapour deposition, plasma spraying) and chemical methods (sol–gel technique, hydrothermal, chemical precipitation, electrodeposition). At present, the most extensively used method to prepare layers is the plasma spraying [11,12,13]. This method can provide well adherent, homogeneous, and crystalline coatings which are efficient and steady. However, this technique requires expensive equipment, maintenance, high temperature, and it is so called the line-of-sight technique. On the contrary, electrochemical deposition is a versatile technique that has been used widely in biomedical applications [14,15,16]. Using the electrochemical process to prepare coatings is more cost effective compared to the above mentioned physical deposition methods and the processing temperature is significantly lower. The properties of the layers can be easily adjusted, a relatively homogenous layer can be achieved on complex-shaped, three-dimensional materials (non-line-of-sight technique) and a high purity of deposits with an optimum particle size can be obtained [17,18,19]. The electrochemical deposition can be performed by the direct and pulse current. Applying the pulse current over the direct current also enables the improvement of properties and structures of coatings by adjusting the appropriate deposition parameters [20,21]. The short current pulses prefer nuclei generation to growth, thus coatings with a smaller grain size can be deposited which can change the corrosion properties of samples [22]. The coatings’ morphology, and, as a consequence, their chemical and biological properties can be also altered by co-deposition of calcium phosphates with other metallic ions or ceramics [23,24,25]. The incorporation of different elements into CP coatings can be performed by electrochemical co-deposition from electrolytes containing the substituting elements in appropriate concentrations [26,27]. Several studies have discussed the advantage of Zn substitution for Ca in hydroxyapatite [28,29]. A trace amount of Zn, around 0.012–0.250 wt% in the human bone, can stimulate new bone formation and also inhibits osteoclastic bone resorption [30,31]. Another recent review discusses that the zinc content is beneficial for osteoclastogenesis [32]. The higher Zn content of coating exhibits an antimicrobial property and hence prevents and minimizes the bacterial adherence [33,34]. On the other hand, Zn deficiency might cause prolonged bone healing and even osteoporosis [35]. Sutha et al. [36] prepared Zn incorporated hydroxyapatite (HAp) coatings on a stainless steel substrate via the spin coating method and studied their antimicrobial properties. Enhanced antimicrobial characteristic of Zn-HAp coating was revealed with a Zn concentration of over 6 mol%. Silver ions are also broad-spectrum antibiotics. The preparation and application of silver substituted calcium phosphate coatings are exceptionally widespread and investigated with regard to its antibacterial properties [37,38,39,40,41,42,43]. However, a proper silver concentration has to be chosen to adjust the biocompatibility and antibacterial characteristics. Lu et al. [41] carried out biocompatibility tests by the Alamar Blue assay on osteoblast cells grown on different samples. They have found that pure HAp layers possessed the highest proliferation rate, followed by 0.5 and 1 HAp/Ag after 3 days of cell culture. Kumar et al. [44] recently developed electroactive mesoporous silver-doped bio-ceramics (MBCs) for medical use. These ceramics were synthetized via the sol-gel method. The conductive MBCs with a silver-content had better antibacterial effect and also improved the cell proliferation rate. The cell viability studies have also proven that there was no noticeable cytotoxic response for 2, 5, 7.5, and 10 mol% Ag-containing MBCs. While Lenis et al. [45] prepared a multi-layered, antibacterial silver containing calcium phosphate coatings onto Ti6Al4V alloy with radio frequency magnetron sputtering, in which the silver content was 4.2 at%. They found that the bacterial growth inhibition at low silver concentration on *P. aeruginosa* and *S. aureus* strains was efficient. In addition, the biocompatibility tests showed that there was no toxic effect of the silver-CaP samples on Saos-2 osteoblast cells.

Although ionic substituted calcium phosphate coatings have been widely studied so far, the effects of these substitutions on the morphology of CP crystals and the elemental distribution of these elements within the coatings have not yet been fully investigated. Moreover, the number of reports on the co-deposition of calcium ion with different bioactive ions by the pulse current is still limited. The time of current impulses applied in our work is in the millisecond range which is followed by a relaxation time. Our study is also important to confirm that the ionic substitution influences the biodegradable properties of layers in physiological solutions.

In this paper, we aim to present and compare the morphology characteristics of ionic substituted CP coatings that were co-deposited onto commercially available titanium alloy (Ti6Al4V) by the pulse current in a millisecond range. The biodegradability of these coatings is investigated through corrosion measurements, while their cytotoxicity has been tested by cell viability tests.

## 2. Materials and Methods

### 2.1. Sample Preparation by Pulse Current Deposition

In the electrodeposition process, the base electrolyte was 0.49 M Ca(NO_3_)_2_·4H_2_O, 0.29 M NH_4_H_2_PO_4_, and 10 mL/L H_2_O_2_ (30%) (VWR International Ltd. Reag. Ph. Eur.). For the electrodeposition of ionic substituted calcium phosphate layers, 10^−3^ M AgNO_3_ and 10^−2^ M ZnNO_3_ (VWR International Ltd. AnalaR Normapur) chemicals were also added to the base electrolyte. The pH of the electrolyte varied between 4.0 and 4.5. The deposition process was done in a two-electrode cell at 70 °C, for 10 min using the IGTV-4i/6t type pulse current generator (BUTE, Budapest, Hungary). The anode was a platinum sheet and the cathode was the metallic implant disc (titanium alloy, Ti6Al4V discs with 19 mm in diameter, and 1 mm in thickness, Protetim LtD., Hódmezővásárhely, Hungary). The deposition parameters: *t*_on_: 5 ms, *t*_off_: 5 ms, and current density: 400 mAcm^−2^.

### 2.2. Morphological and Structural Characterization

The morphological characteristics of the layers have been studied by SEM–EDX (Hitachi SU8230, Jeol 8230, Hitachi Group, Tokyo, Japan) and TEM (FEI Tecnai TF2, Thermo Fisher Scientific, Hillsboro, OR, USA).

The crystal structures of the samples have been investigated by X-ray diffraction. XRD diffractograms have been recorded at room temperature by Bruker AXSD8 Discover diffractometer (Cu Kα radiation source) equipped with Göbel mirror and GADDS 2D detector system (Bruker, Wien, Austria). The operation parameters of equipment were 40 kV and 40 mA. The diffraction patterns have been collected over a 2θ range from 10° to 80° with an 1°/min step by flat plane geometry. The Diffrac.Eva V5.2 software was used to evaluate the XRD patterns and to determine the crystallite phases.

### 2.3. Corrosion Measurements

Potentiodynamic polarization measurements have been performed by Zahner IM6e potentiostat/galvanostat (Zahner, Kronach Germany) in order to test the corrosion characteristic and biodegradability of coatings. In all corrosion measurements, a three-electrode cell was used, where the working electrode was a metallic implant disc with and without coatings, the reference electrode was Ag/AgCl/KClsat. electrode, and the counter electrode was the platinum net. The potentiodynamic polarization curves were recorded with a 1 mV/s scanning rate in the conventional Ringer’s solution: NaCl: 9.00 g/L, NaHCO_3_: 0.20 g/L, KCl: 0.43 g/L, CaCl_2_: 0.24 g/L, pH: 7.95. The pH was adjusted with 1 M HCl and 1 M NaOH solutions.

### 2.4. Cytotoxicity Measurements

For cell viability measurements, the investigated samples were put in a 24-well microtiter plate (MTP). One milliliter of cell suspension (MG-63 Cell Line human, Merck KGaA, Darmstadt, Germany, 10,000 cells/mL) was seeded onto the surface of each sample. The same quantity of cells in a growth medium was applied as the control with no samples. The culture medium was removed from the 24-well culture plate after 7 and 14 days of cultivation, the cells were washed using a sterile phosphate-buffered saline (PBS). Then, 1 mL of DMEM (Dulbecco’s Modified Eagle Medium, containing a 1% WST-8 tetrazolium salt (2,3,5-triphenyl-2H-tetrazolium chloride) reagent was added to each well and was incubated for 3.5 h. During the incubation time, the viable cells turned the WST-8 reagent into a water soluble, yellow formazan dye. Spectrophotometric measurements were executed after incubation on the coloured products. The specific absorbance of formazan dye (at 450 nm) in the MTP was obtained with an ELISA plate reader (PHomo Autobio Anthos Mykrosystem GMbh, Friesoythe, Germany). Absorbance values are presented by the mean value and standard deviation of six replicates of each sample type and were normalized to MG-63 cells growth on a well plate (control = 100%). The absorbance values directly refer to the amount of live cells.

## 3. Results and Discussion

### 3.1. Morphological Characterization

#### 3.1.1. Silver-Added Calcium Phosphate Coating

**Figure 1:**
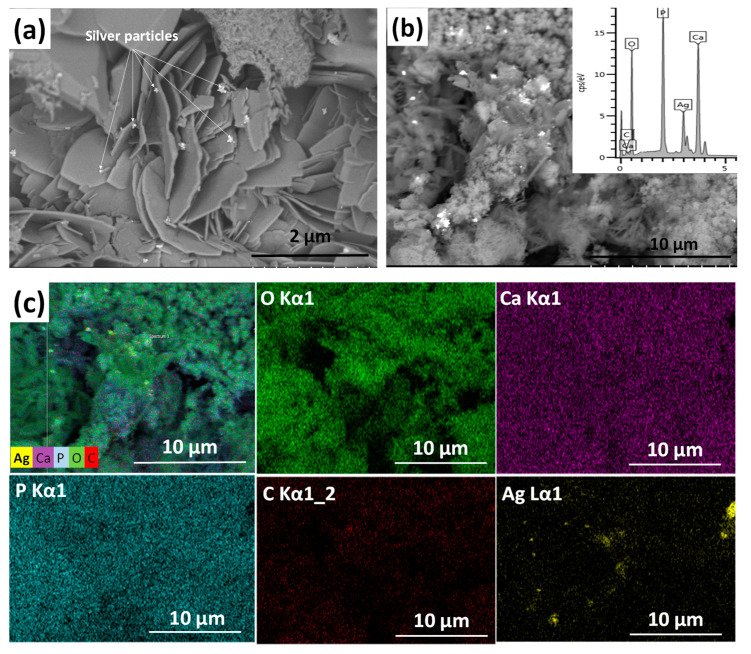
SEM image of silver-containing calcium phosphate (CP) coating. (**a**) Magnification: 20.0 k, working voltage: 2.0 kV, working distance: 4.1 mm (**b**) magnification: 5.0 k, working voltage: 10 kV, working distance: 15.1 mm, and the corresponding map sum EDX spectrum (inset) and (**c**) elemental mapping of silver-containing CP coating: Overlay map; O (Kα1 line); Ca ((Kα1 line); P (Kα1 line); C (Kα1_2 line); Ag (Lα1 line).

Figure 1 shows the morphology and surface characteristics of silver-doped calcium phosphate coating. The silver-containing calcium phosphate layer consists of particles in different, non-uniform shapes. It contains disoriented, small, needle-like, larger plate-like, and also spheroid grains in various sizes.

The silver particles (white spots in Figure 1a) are 100–150 nm in size and they tend to agglomerate into larger flakes (Figure 1b). The elemental mapping reveals the presence of silver, calcium, phosphor, and carbon elements. The carbon signal proves the presence of a carbonated calcium phosphate phase. It is also visible that the distribution of silver element is not homogeneous.

#### 3.1.2. Zinc-Added Calcium Phosphate Coating

**Figure 2:**
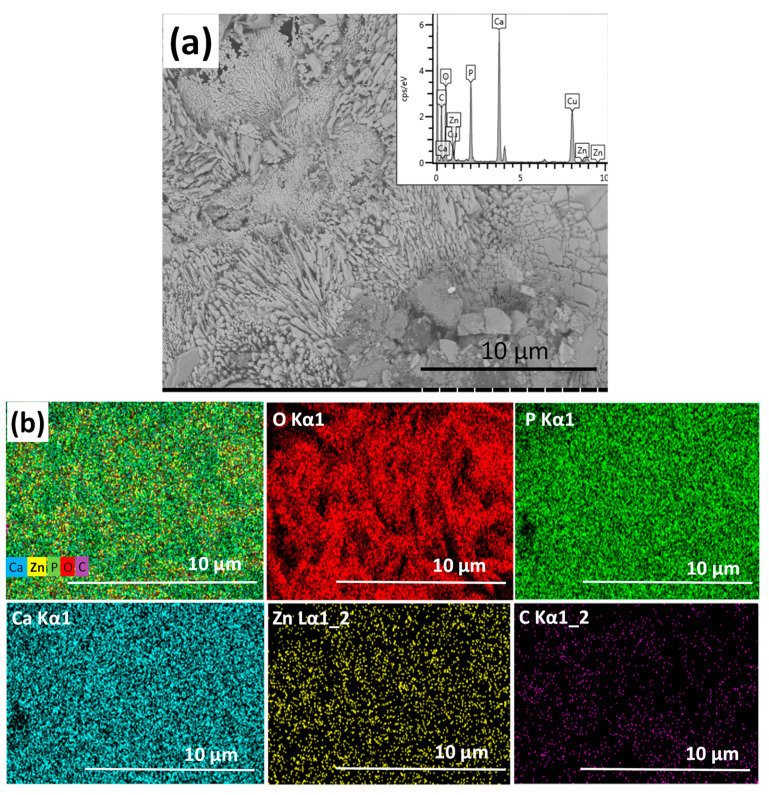
SEM image of zinc-containing CP coating. (**a**) Magnification: 5.0 k, working voltage: 2.0 kV, working distance: 4.1 mm, and the corresponding map sum EDX spectrum (inset) and (**b**) elemental mapping of zinc-containing CP coating.

The zinc-containing calcium phosphate coating consists of large, plate-like agglomerates and blocks in micrometre size (Figure 2). According to the EDX analyses, the zinc content of layer varies between 5–40 at% depending on the site of investigation. This can indicate that different phases are present in the coating, consisting of Ca-rich phosphates and Zn-rich phosphates in different ratios at the different sites of the sample.

The elemental map on Zn-containing CP shows that the distribution of zinc element within the layer is more homogeneous than in the case of silver co-deposition. During the electrochemical deposition process, the zinc ions migrate toward the cathode along with the calcium ions and form deposits of different Ca–Zn phosphate phases. According to Prado da Silva [46], the hydrothermal deposition method allows Monetite (CaHPO4) and Parascholzite (CaZn_2_(PO_4_)_2_(H_2_O)_2_) precipitation onto metallic substrates and produces zinc apatite coatings. They reported that Monetite, brushite, and Parascholzite crystals were grown within the oxide layer on the surface of substrate and zinc addition to the solution impeded in the nucleation and growth processes. They associated this phenomenon to the lower affinity of zinc for oxygen compared to calcium, owing to the lower zinc electronegativity difference, which increased the energy barrier for grain nucleation and growth.

#### 3.1.3. Silver- and Zinc-Added Calcium Phosphate Coating

**Figure 3:**
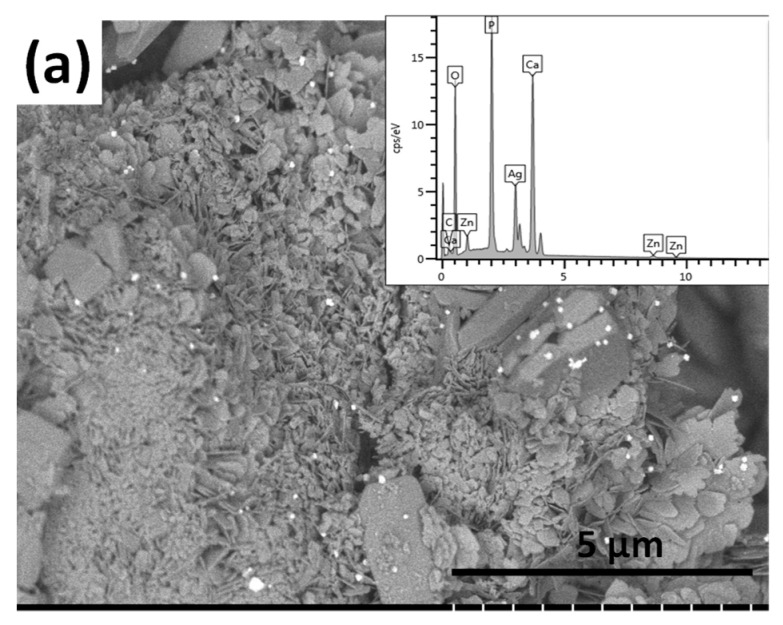

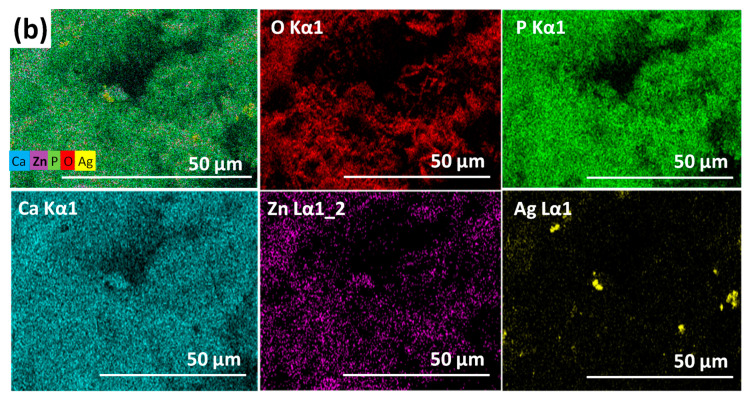
SEM image of silver- and zinc-containing calcium phosphate coating. (**a**) Magnification: 10.0 k, working voltage: 2.0 kV, working distance: 8.0 mm, and the corresponding map sum EDX spectrum (inset) and (**b**) elemental mapping of silver- and zinc-containing CP coating.

As Figure 3 demonstrates, this layer also contains particles in a wide variety of shapes and sizes. It comprises grains with a mixture of heterogeneous, small, needle-like particles in nanometre size and larger, thin, plate-like, and rod-shaped particles in micrometre size. The size of silver particles in this case are similarly between 100 and 150 nm, and they tend to agglomerate. It can also be observed that when silver and zinc particles were added into the layer, they were co-deposited onto the implant surface along with different calcium phosphate phases. Iqbal et al. [47] co-substituted the hydroxyapatite particles with silver and zinc via the microwave-assisted wet precipitation method and investigated the purity of phases, the morphology, elemental composition, and the particle size of samples, as well as their antibacterial effects against Staphylococcus aureus and Escherichia coli. In their experiments, the Zn concentration changed between 2 and 4 wt%, while the silver content was 0.3 wt%. According to the SEM measurements, the zinc and silver addition changed the morphology of the HAp particles. The nanoparticles were round shaped with a diameter of around 102 nm and they tended to agglomerate. The Ag and Zn doped samples showed superior antibacterial properties compared to the pure hydroxyapatite. While Guo et al. [24] prepared hydroxyapatite co-deposited with nano-sized silver and zinc oxide particles with a two-step liquid chemical reduction technique. They used calcium hydroxide and phosphoric acid as precursors for HAp synthesis. Zinc nitrate was the precursor for ZnO particles and the silver ammonia solution to generate silver nanoparticles. The morphological study revealed that most of the HAp nanoparticles were in a rod-shaped form with a diameter of around 40 nm and the spherical-shaped silver nanoparticles, with an average size of 6 nm, were on the surface of HAp nanoparticles. The average size of plate-like ZnO nanoparticles was 23.5 nm.

### 3.2. Transmission Electron Microscopy Study

**Figure 4:**
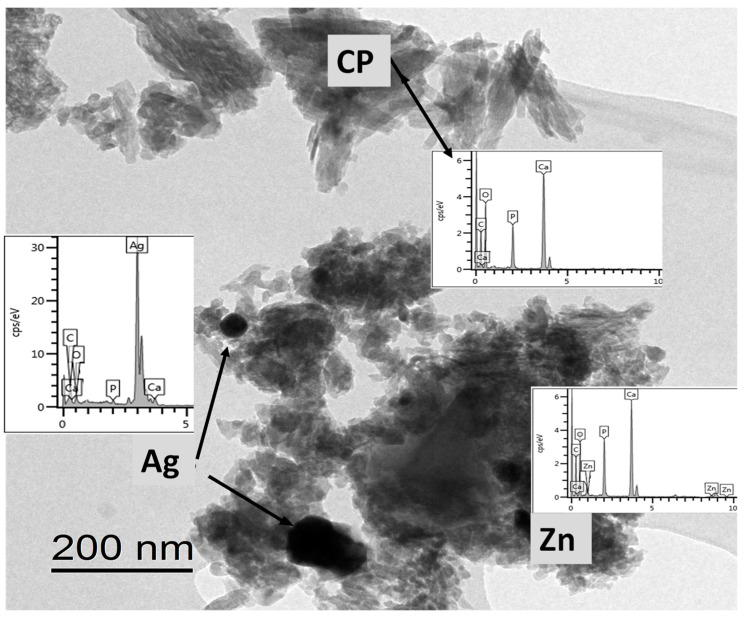
TEM image and EDX elemental analysis of silver and zinc substituted CP powder.

TEM and EDX investigations of silver- and zinc-doped iCP powders, prepared by pulse current co-deposition, are presented in Figure 4. The TEM–EDX measurements have been performed to further examine the form and distribution of different grains in the silver- and zinc-added calcium phosphate sample. Figure 4 reveals that the silver and zinc particles were incorporated in the calcium phosphate crystals. The zinc and silver signal became dominant on different sites of the sample, which indicate the presence of these particles along with the calcium phosphate crystals. It is also visible that the silver particles are in the form of larger grains in size around 50–100 nm.

Syukkalova et al. [48] synthesized calcium phosphate nanoparticles with a novel hydrothermal method and investigated the morphological parameters of the samples. In their work, they used different kinds of surfactants, such as PVP, SDS, and calcium stearate to study their effect on the morphological parameters of nanoparticles. They found that the addition of surfactants to the solutions did not cause any significant change in the phase composition, however, they greatly affected the formation processes of the nanoparticles, as well as the sizes and shapes of the grains. The size of particles derived from TEM measurements varied between 10 and 34 nm in thickness and 50–140 nm in length depending on the type of surfactant.

### 3.3. XRD Measurements on Ionic Substituted Calcium Phosphate Coatings

**Figure 5:**
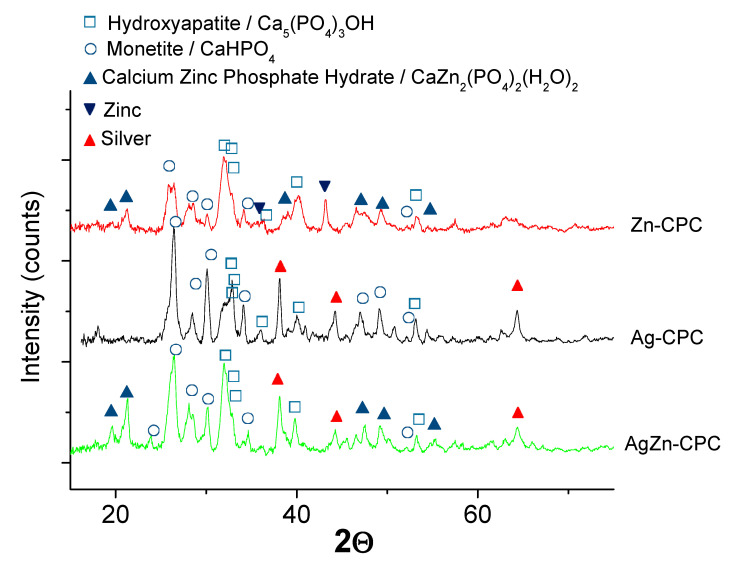
XRD spectra recorded on silver- and zinc-added calcium phosphate powder.

XRD patterns of iCP layers are demonstrated in Figure 5. Patterns were recorded on Ag and/or Zn added CP layers. The characteristic peaks can be distinguished as a mixture of calcium phosphate phases such as hydroxyapatite (HAp, Ca_5_(PO_4_)_3_(OH), JCPDS76-0694) and Monetite (DCP, CaHPO_4_, JCPDS89-5969). The characteristic peaks of HAp component are at *d* = 2.814 Å and *d* = 2.719 Å, while the DCP component’s main peaks appear at *d* = 3.349 Å and *d* = 2.726 Å. In the case of Ag-added calcium phosphate phases, the most intensive peak of Ag (111) are found on the spectra (Ag, JCPDS89-3722) at *d* = 2.358 Å. In this case, the silver grains are present in a metallic form in the layer. The XRD patterns of Zn and Ag/Zn substituted calcium phosphate layer show that the Zn particles are present in two main phases: Metallic Zn (Zn, JCPDS87-0713) and CaZn_2_(PO_4_)_2_(H_2_O)_2_ (Parascholzite, JCPDS86-2372). The reflection of the main peak of metallic Zn is at *d* = 2.091 Å and for Parascholzite is at *d*=4.156 Å. The ratio of different phases, calculated by the Diffrac.eva software V5.2, was as follows in the silver and zinc substituted CP coating: HAp:DCP:Parascholite:Ag = 39.7:20.1:36.8:3.4. In the Ag and Zn substituted calcium phosphate coating, the metallic zinc was not detectable, which can imply the favoured formation of calcium zinc phosphate hydrate phase at low Zn ion concentrations in the electrolyte during deposition. This result is in agreement with other research works [29,46] where the Zn incorporation in the form of Parascholzite is proved by XRD measurements in which its main peaks can also be found, in addition to Monetite’s and Brushite’s (CaHPO_4_ and CaHPO_4_·2H_2_O).

Ren et al. [28] reported line shifting to higher 2Θ values owing to the substitution of larger sized Ca^2+^ (100 pm) ions with smaller sized Zn^2+^ (74 pm) ions.

### 3.4. Corrosion Measurement on Ionic Substituted Calcium Phosphate Coatings

**Figure 6:**
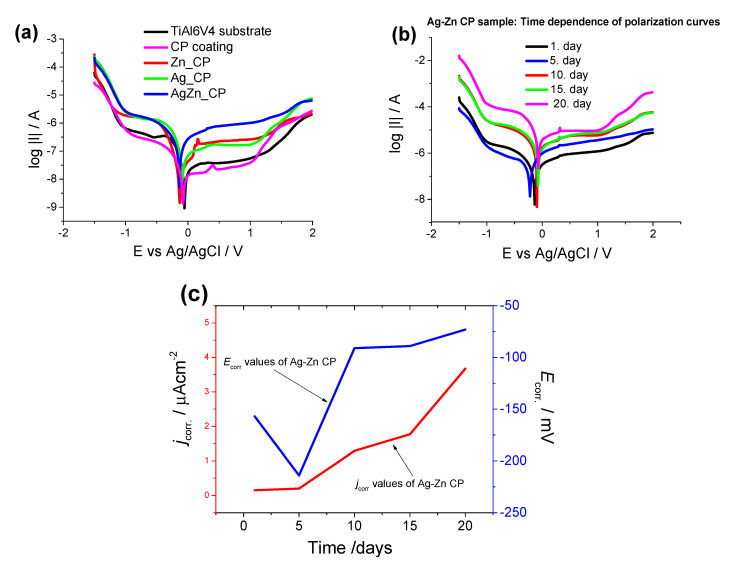
Polarization curves recorded on different samples after 5 days of immersion. (**a**) Time-dependence of potentiodynamic polarization curves recorded on Ag and Zn substituted CP coating, (**b**) as well as the *j*_corr_. (red line) and *E*_corr._ (blue line) values derived from potentiodynamic curves (**c**). The curves were recorded in conventional Ringer’s solution at 37 °C with a potential scan rate of 1 mv s^−1^.

Potentiodynamic polarization measurements have been performed to compare the degradation characteristic of titanium substrate, of pure CP and of iCP coatings in Ringer’s solution (Figure 6). The polarization scan rate was 1 mV s^−1^. The potentiodynamic curves were evaluated to attain the corrosion parameters.

It is visible that the titanium substrate and the CP coating have the lowest corrosion currents (0.2–0.4 µAcm^−2^) [49] and the highest corrosion resistance, while the highest corrosion rate and lowest corrosion resistance belong to the silver and zinc substituted CP layer. The uncoated substrate and the CP sample have a nobler corrosion potential than that of ionic substituted CP coatings (Figure 6a). The *E*_corr_ values of Ag and Zn substituted CP coating decreased significantly in the early stage of immersion then reached a quasi-steady-state value after 10–15 days of immersion (Figure 6b,c). This phenomenon confirms the gradual passivation of the surface. Supposedly, the reason for this passivation is the corrosion products formed during immersion that can penetrate into the pores of the coating, hence inhibiting the dissolution processes.

The corrosion currents of silver and zinc substituted CP gradually increase over time from 1 to 5 µAcm^−2^ (Figure 6c), which can prove the biodegradable characteristic of iCP coating. The highest *j*_corr_ values were obtained in the case of Ag and Zn substituted CP layer indicating that the rate of the dissolution processes was higher than in the case of pure CP and uncoated implant. The iCP coating exhibited the greatest tendency towards corrosion. In a recent work, Jaiswal et al. [50] studied the effect of shape and size of bioactive hydroxyapatite on the mechanical and biodegradation properties of the magnesium-based composite. The HAp layer increased the corrosion resistance of magnesium alloy substrate and the measured corrosion currents were 0.06–0.09 mAcm^−2^ and were dependent on the morphology of the HAp coating. While Kwok et al. [51] studied the corrosion properties of HAp coating prepared by electrophoretic deposition onto a Ti6Al4V substrate. According to their results, the measured corrosion currents in Hanks’ solution varied between 0.4 and 0.9 µAcm^−2^, depending on the morphology of the HAp powder. There are also research works on studying the corrosion characteristic of ionic doped CP coatings [34,52,53,54,55,56,57,58,59,60]. Iqbal et al. [34] reported the biodegradability properties of zinc-doped hydroxyapatite−zeolite/polycaprolactone composite coating, prepared by dip coating onto a magnesium substrate. The measured corrosion currents in their case changed between 10 and 25 µAcm^−2^ in Kokubo’s SBF solution. According to their measurements, the higher Zn content in HAp increased the corrosion resistance of the samples. Gopi et al. [52] prepared Sr, Mg, and Zn doped calcium phosphate coatings on an HELCDEB-treated titanium by the pulse current deposition method. In their case, the samples with a surface treatment exhibited higher *E*_corr_ and *j*_corr_ values than the untreated titanium sample. The *j*_corr_ values varied between 0.4 and 0.22 µAcm^−2^. In other research work, Zhong et al. [53] reported that the corrosion currents of electrophoretically deposited HAp and zinc substituted HAp coating with a 5 wt% Zn content to be 1.45 and 0.58 µAcm^−2^, respectively in the SBF solution. They concluded that the Zn-HAp composite coating possessed better corrosion resistance than the HAp composite coating.

### 3.5. Cell Viability Measurements

**Figure 7:**
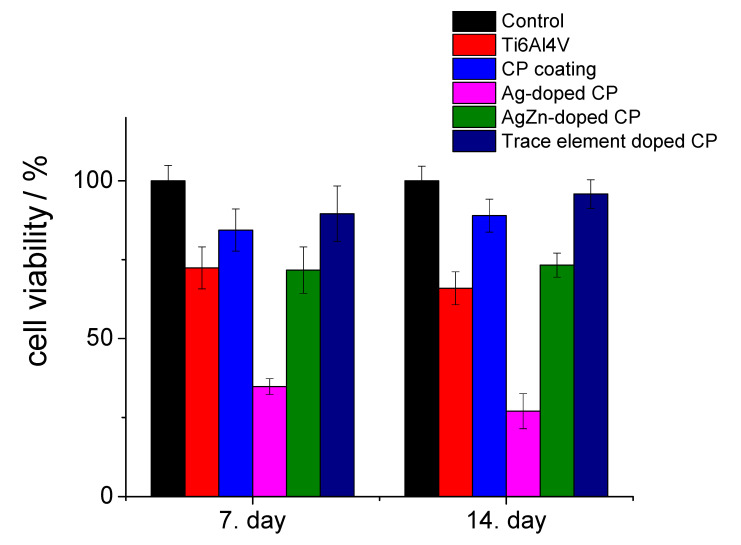
Cell viability percentage on the investigated samples compared to the positive control. Positive control: MG-63 cells were grown in well plates with no samples. Results are demonstrated by the mean value and standard deviation of six replicates of each sample and were normalized to MG-63 cells growth on a well plate (control = 100%).

Figure 7 shows the viability percentages of cells (MG-63 osteoblast cell line) seeded on the substrate material and different CP coatings compared to the control sample. Along with the substrate, four different types of coatings were investigated: (1) Pure CP (2) silver-added CP, where the silver concentration within the coating was relatively high (between 3–10 at%), (3) Ag- and Zn-added CP coating, where the percentages of doping elements were around 40 at%, according to the XRD measurements; (4) trace-element doped CP coating, in which the concentration of all doping elements was below 5 at% in total.

It is visible that in all culture periods the trace element doped CP possessed the highest cell viability percentages after 7 days (above 89%), while after 2 weeks they increased to around 95% compared to the positive control. The cell viability percentages were 84% and 89% after 7 days and 2 weeks of culture for the CP sample, respectively. We obtained similar values (71% and 73%) for the Ag/Zn-CP sample, where the silver concentration was very low. However, in the case of silver doped sample, we measured relatively low cell viability values (35% and 27%) owing to the high silver content. In the case of titanium substrate without a coating, the cell viability was 72% at the 7th day and it decreased to 65% at the 14th day. After 2 weeks of culture in a DMEM medium, the difference between the cell viability on CP and on trace element doped CP samples become more significantly higher than those for the uncoated substrate, revealing the good biocompatible characteristic of this coating. It is also visible that the trace element substitution improved the biocompatibility of the sample. In addition, it is well known that hydroxyapatite layers can promote the attachment and growth of osteoblastic cells due to their excellent hydrophilic characteristic [61,62].

Numerous aspects exist that can affect the viability and metabolic activity of MG 63 cells, such as the composition, morphology, and structure of layers. Several research works have proven that the excessive silver content in the layer can provide good antimicrobial properties to the layers, but, parallelly, it also has a negative effect on the viability and metabolism of cells and can also cause mitochondrial dysfunction [63,64,65]. However, on the contrary, Thrivikraman et al. [64] claimed in their review that the addition of 10% Ag into Ag-HAp composites provided an antibacterial effect against *E. coli*, without deteriorating the in vitro cytocompatibility of the HAp. There are also research works studying the biological response of zinc incorporation into HAp phases [66,67,68]. Ortiz et al. [66] reported that the cell viability percentages of human osteoblast cells seeded on Zn-HAp layers were around 80% in comparison with the control group and they also measured the enhanced cell proliferation rate compared to the HAp coating. They also claimed that the cells responded better to the Zn-HAp coating thus confirming their biocompatibility and ability to support cell proliferation. It is also proven that the Zn addition (around 9 at%) has a beneficial effect on promoting bone growth when used as a bioactive coating on implant materials [68].

## 4. Conclusions

The effects of ionic substitution on the morphology, structure, and thus the biocompatibility properties of calcium phosphate coatings have been studied. The silver-containing calcium phosphate layer contained particles in different, non-uniform shapes: Disoriented small needle-like, larger plate-like, and also spheroid grains in various sizes. The zinc-containing calcium phosphate coating consisted of large, plate-like agglomerates, and blocks in micrometre size. The silver and zinc substituted layer also contained particles in a wide variety of shapes and sizes. The size of silver particles was between 100 and 150 nm, and they tended to agglomerate. The Zn particles were present in two main structures: Metallic Zn and Parascholzite. The corrosion measurements revealed that the corrosion currents gradually increased over time, which could prove the biodegradable characteristic of the iCP coating. The highest *j*_corr_ values were obtained in the case of silver and zinc doped CP coating. The cell viability measurements might indicate that the concentrations of doping elements have an effect on the biocompatibility of coatings, therefore, choosing the appropriate concentrations and ratios of doping elements is essential for biomedical applications.
